# Editorial: Novel ligands and receptors in endocrine disorders

**DOI:** 10.3389/fcell.2023.1218271

**Published:** 2023-05-26

**Authors:** Huashan Zhao, Juxue Li, Stig Ove Bøe, Rujuan Zuo, Peng Zhu

**Affiliations:** ^1^ Center for Energy Metabolism and Reproduction, Shenzhen Institute of Advanced Technology, Chinese Academy of Sciences, Shenzhen, China; ^2^ State Key Laboratory of Reproductive Medicine and Offspring Health, Nanjing Medical University, Nanjing, Jiangsu, China; ^3^ Department of Microbiology, Oslo University Hospital, Oslo, Norway; ^4^ Laboratory for Stem Cell Dynamics, Department of Microbiology, Division of Laboratory Medicine, Oslo University Hospital, Rikshospitalet, Oslo, Norway; ^5^ Department of Maternal, Child and Adolescent Health, School of Public Health, Anhui Medical University, Hefei, China

**Keywords:** ligands, receptors, endocrine disorders, reproductive endocrinology, environmental endocrine disrupting chemicals

Abnormal interactions between ligands and receptors can promote various endocrine disorders. However, due to the complexity of endocrine systems and physiological processes, deciphering mechanisms for the diagnostic and treatment of these diseases remains a challenge. We thank authors from various research fields for their invaluable contributions to this Research Topic. The papers collected in this topic mainly encompass reproductive endocrine disorders, thereby enriching our knowledge on endocrine regulation involving well-known ligands and receptors in other systems. These identified targets shed light on further investigation of their mechanisms.

When an endogenous ligand binds to its receptor in the endocrine tissue cells, the ligand-receptor systems play an imperative role in regulating various physiological functions. Zhang et al. discovered that the chemerin-CMKLR1 system could regulate placental development and vascular remodeling during early pregnancy. Another Zhang et al. provided a comprehensive understanding of osteoprotegerin-RANK in endocrine regulation, reviewing the roles of osteoprotegerin in endocrine and metabolic disorders through the RANKL/RANK signaling. Furthermore, G protein-coupled receptors (GPCRs) family, as potential targets, contain many receptor candidates involved in endocrine disorders. For instance, Yin et al. reviewed the role of secretin/adhesion (Class B) GPCRs in placental development and preeclampsia. Lin et al. reviewed the emergent roles of GPR158 in regulating the endocrine system. And Luo et al. reviewed the effects of GPER on age-associated memory impairment induced by decreased estrogen levels. These three reviews highlight the multifaceted functions of GPCRs in endocrine disorders. In addition, Huang et al. discovered that the membrane ion channel protein, AQP8, could regulate ovarian secretion by modulating granulosa cell autophagy, suggesting that endocrine cells are not regulated solely by the ligand-receptor system.

Besides, receptors in endocrine cells can also be activated by exogenous ligands, including environmental endocrine disrupting chemicals (EDCs) and pharmaceutical molecules. In the field of exposure science, increasing studies focus on EDCs and their potential roles in endocrine disorders. As ligand analogs, EDCs disrupt endocrine hormone functions, leading to endocrine disorders and abnormal tissue development. Zhang et al. found that di-(2-ethylhexyl) phthalate (DEHP) exposure induced liver injury by promoting ferroptosis via downregulation of GPX4 in pregnant mice. Additionally, Zeng et al. reviewed the impact of early-life exposure to DEHP in children with endocrine disorders. Similarly, pharmaceutical molecules could be used to treat endocrine disorders by activating corresponding receptors. Kong et al. reviewed recent advances in our understanding of the effects of *Salvia miltiorrhiza* active compounds on placenta-mediated pregnancy complications, indicating that many receptors play essential roles in the multiple endocrine events during pregnancy. These traditional Chinese medicine monomers could directly activate the corresponding receptors and improve the clinical symptoms, although the mechanism remains largely unknown.

Regarding research methods, Qu et al. established the three-dimensional visualization of mouse endometrial remodeling after superovulation, which is a valuable attempt. The response of the endometrial tissue to endocrine disturbance can be observed using the uterine 3D reconstruction technology.

In summary, this Research Topic aims to explore target ligands and receptors that regulate endocrine disorders, with a particular emphasis on the emerging roles of EDCs. Mechanism research, focusing on ligands or receptors, is still ongoing. It is hopeful that the discovery of more suitable drug targets, especially GPCRs, would aid in the treatment of endocrine disorders.

